# Adhärenz digitaler Interventionen im Gesundheitswesen: Definitionen, Methoden und offene Fragen

**DOI:** 10.1007/s00103-021-03415-9

**Published:** 2021-09-24

**Authors:** Sven Kernebeck, Theresa Sophie Busse, Jan Peter Ehlers, Horst Christian Vollmar

**Affiliations:** 1 Lehrstuhl für Didaktik und Bildungsforschung im Gesundheitswesen, Fakultät für Gesundheit, Universität Witten/Herdecke, Pferdebachstraße 11, 58448 Witten, Deutschland; 2 Medizinische Fakultät, Abteilung für Allgemeinmedizin, Ruhr-Universität Bochum, Bochum, Deutschland

**Keywords:** Adhärenz, Digitale Interventionen, Wirksamkeit, M‑Health, E‑Health, Digitale Gesundheitsanwendungen, Adherence, Digital interventions, Effectiveness, M‑Health, E‑Health, Digital health apps

## Abstract

Viele digitale Interventionen sind auf die Mitwirkung ihrer Nutzer*innen angewiesen, damit sie eine positive Wirkung erzielen können. In verschiedenen Bereichen ist zu beobachten, dass die Anwendung digitaler Interventionen durch Nutzer*innen oftmals nach einer kurzen Zeit reduziert oder in Gänze eingestellt wird. Dies wird als einer der wesentlichen Faktoren angesehen, der die Wirksamkeit digitaler Interventionen einschränken kann. In diesem Zusammenhang gewinnt das Konzept der Adhärenz (Einhalten therapeutischer Vorgaben) bei digitalen Interventionen zunehmend an Bedeutung. Definiert wird Adhärenz bei digitalen Interventionen etwa als „the degree to which the user followed the program as it was designed“ („Ausmaß, in dem die Nutzer*innen die Software so verwenden, wie sie konzipiert wurde“). Dies wird auch oftmals mit „intended use“ oder „use as it is designed“ umschrieben („bestimmungsgemäße Verwendung“ bzw. „Verwendung, wie es konzipiert wurde“). Jedoch finden sowohl die theoretisch-konzeptionelle als auch die praktische Auseinandersetzung hinsichtlich der Adhärenz bei digitalen Interventionen noch eine zu geringe Berücksichtigung in der Forschung.

Ziel dieses narrativen Übersichtsartikels ist es, das Konzept der Adhärenz bei digitalen Interventionen näher zu beleuchten und von verwandten Konzepten abzugrenzen. Zudem wird diskutiert, mit welchen Methoden und Messgrößen die Adhärenz operationalisiert werden kann und welche Prädiktoren die Adhärenz positiv beeinflussen. Weiterhin wird auf die Dosis-Wirkungs-Beziehung bei der Anwendung digitaler Interventionen eingegangen und auf Faktoren, die die Adhärenz positiv beeinflussen können. Abgeschlossen wird mit einer ethischen Betrachtung der Thematik.

## Abstract (en)

Many digital interventions rely on the participation of their users to have a positive impact. In various areas it can be observed that the use of digital interventions is often reduced or fully discontinued by the users after a short period of time. This is seen as one of the main factors that can limit the effectiveness of digital interventions. In this context, the concept of adherence to digital interventions is becoming increasingly important. Adherence to digital interventions is roughly defined as “the degree to which the user followed the program as it was designed,” which can also be paraphrased as “intended use” or “use as it is designed.” However, both the theoretical–conceptual and practical discussions regarding adherence to digital interventions still receive too little attention.

The aim of this narrative review article is to shed more light on the concept of adherence to digital interventions and to distinguish it from related concepts. It also discusses the methods and metrics that can be used to operationalize adherence and the predictors that positively influence adherence. Finally, needs for action to better address adherence are considered critically.

## Hintergrund

Im Gesundheitswesen gibt es verschiedene digitale Interventionen, die über digitale Endgeräte, beispielsweise Smartphones, Tablet- und Desktop-Computer oder Wearables, genutzt werden können [1]. Ein wesentliches Ziel digitaler Interventionen im Gesundheitswesen ist die positive Beeinflussung gesundheitsbezogener Outcomes [2]. Dies umfasst etwa die Beeinflussung verhaltensbezogener Eigenschaften, wie etwa die Reduktion des Rauchens [3], die Steigerung der körperlichen Aktivität [4] oder das Management chronischer Krankheiten [5]. Digitale Interventionen sind häufig komplex, da sie multiple, interagierende Komponenten beinhalten, mit denen verschiedene Ziele erreicht werden sollen. Hierzu zählen die Bereitstellung von Gesundheitsinformationen, das Überwachen und die Dokumentation von Vitalparametern oder die Verbesserung der Kommunikation zwischen Nutzer*innen und Versorger*innen [1].

Obwohl die Potenziale digitaler Interventionen meist hoch eingeschätzt werden, finden sich in bestimmten Bereichen hinsichtlich ihrer Wirksamkeit heterogene Ergebnisse, die auf eine eingeschränkte Effektivität hinweisen [6]. Dies lässt sich etwa im Bereich Essstörungen [7], Diabetes [8] oder bei der Prävention von Übergewicht feststellen [9]. Positive Ergebnisse werden demgegenüber etwa bei dem Selbstmanagement der Hypertonie [10] oder bei dem Selbstmanagement psychischer Krankheiten [11] berichtet. Die Gründe für eine geringe Wirksamkeit sind vielfältig und reichen von einer eingeschränkten theoretischen Fundierung, einem geringen Grad an Standardisierung bei Evaluationen [12], kurzen Beobachtungszeiträumen [3], hohen Drop-out-Raten [13] bis hin zu einer hohen Variabilität von Interventionskomponenten [10].

Ein weiterer Grund, der oft zu wenig beachtet wird, ist die eingeschränkte Adhärenz der Nutzer*innen bei der konkreten Anwendung [2]. Im Kontext digitaler Intervention lässt sich Adhärenz als *aktive *oder* effektive Anwendung* oder als Intensität der Anwendung entsprechend den Vorgaben der Entwickler*innen beschreiben [14]. Bei medikamentösen Interventionen ist das Konzept der Adhärenz seit vielen Jahren etabliert und bekannt für seine Bedeutung für deren Effektivität [15].

Das Interesse an der Konzeptualisierung der Adhärenz ist in den vergangenen Jahren gestiegen, jedoch bestehen verschiedene Probleme in der aktuellen Forschungspraxis: In der Literatur wird das Konzept der Adhärenz bei digitalen Interventionen weder präzise konzeptualisiert noch eindeutig von verwandten Konzepten, wie etwa dem „Engagement“ (Erläuterung folgt) abgegrenzt [16]. Es besteht zudem Uneinigkeit darüber, mit welchen Methoden und Messgrößen die Adhärenz operationalisiert und evaluiert werden sollte [14]. Ebenso werden in Studien keine oder nur unzureichende Kriterien definiert, die eine gute von einer schlechten Adhärenz unterscheiden, oder Faktoren, die die Adhärenz der Nutzer*innen positiv oder negativ beeinflussen [2]. Zudem findet kein transparentes Reporting dieser Messgrößen statt, was die Beurteilung und Reproduktion der Ergebnisse limitiert. Es ist außerdem nicht hinreichend belegt, welche „Dosis“ für verschiedene Anwendungsfälle notwendig ist, um Outcomes positiv zu beeinflussen [14].

Ziel dieses Artikels ist es, die hier skizzierten Probleme in der aktuellen Forschungspraxis zum Konzept der Adhärenz bei digitalen Interventionen zu erörtern und die Bedeutung für eine bessere methodische Adressierung aufzuzeigen. Wenngleich die Diskussionspunkte in diesem Artikel die Adhärenz bei digitalen Interventionen übergeordnet thematisieren, also unabhängig von der Art der Intervention oder der verwendeten Technologie, lassen sie sich auch auf die Evaluation von smartphonebasierten digitalen Interventionen übertragen. Folglich sind die hier erörterten Inhalte bei der Evaluation und Evidenzbasierung digitaler Gesundheitsanwendungen (DiGA) ebenso relevant.

Zunächst wird daher das Konzept der Adhärenz im Kontext digitaler Interventionen erörtert und von verwandten Konzepten abgegrenzt. Anschließend werden Determinanten, die die Adhärenz beeinflussen, sowie mögliche Messgrößen zur Operationalisierung beschrieben. Weiterhin wird auf die Dosis-Wirkungs-Beziehung bei der Anwendung digitaler Interventionen eingegangen und auf Faktoren, die die Adhärenz positiv beeinflussen können. Abgeschlossen wird mit einer ethischen Betrachtung der Thematik.

## Attrition, Adhärenz und Engagement bei digitalen Interventionen

Wie bereits beschrieben, besteht bei digitalen Interventionen zu gesundheitsbezogenen Themen eine hohe Wahrscheinlichkeit, dass ein Anteil der Nutzer*innen die Interventionselemente nicht wie von den Entwickler*innen vorgesehen anwendet [2]. Dies kann zu einer eingeschränkten Wirksamkeit und möglicherweise zur Einstellung der Nutzung führen [17, 18]. Dieses Phänomen wurde bereits im Jahr 2005 durch Eysenbach als Law of Attrition (sinng. „Gesetz der Erschöpfung“) benannt und als eines der zentralen Probleme bei Studien zu digitalen Interventionen hervorgehoben [18]. Eysenbach unterscheidet zwei Arten von Attrition/Erschöpfung. Das gänzliche Einstellen der Anwendung bezeichnet er als „nonusage attrition“ und das Herausfallen aus Studien als „dropout attrition“ [18].

Ergänzend zu dem *Law of Attrition* hat sich das Konzept der Adhärenz bei digitalen Interventionen herausgebildet. In der Literatur finden sich hierzu unterschiedliche Definitionen. Adhärenz bei digitalen Interventionen wird etwa als „the degree to which the user followed the program as it was designed“ definiert („Ausmaß, in dem die Nutzer*innen die Software so verwenden, wie sie konzipiert wurde“; [17]). Dies wird oftmals mit „intended use“ oder „use as it is designed“ umschrieben („bestimmungsgemäße Verwendung bzw. Verwendung, wie es konzipiert wurde“; [19]). Hiermit wird etwa ein Bezug zur Adhärenz von Medikamenten und Therapiemaßnahmen hergestellt.

In einer weiteren Definition wird die Adhärenz bei digitalen Interventionen als „… the extent to which individuals experience the content of the Internet intervention“ definiert („Ausmaß, in dem Individuen/Nutzer*innen die Inhalte einer internetbasierten Intervention erleben/wahrnehmen“; [20]). Dies hebt im Gegensatz zu „use as it is designed“ oder „intended use“ zudem die gemachten Erfahrungen der Nutzer*innen hervor.

Eine Präzisierung wird von Sieverink et al. vorgenommen. Laut ihrer Definition erfordert die Bestimmung der Adhärenz 3 zentrale Elemente: (1) das Nutzungsverhalten der Nutzer*innen zu messen, (2) die Operationalisierung der intendierten Anwendung zu definieren sowie (3) eine empirische und theoretische Begründung der intendierten Anwendung darzulegen [2].

Abzugrenzen von der Adhärenz ist das Konzept des „Engagements“ (im Sinne von „Einbindung“), obwohl beide Begrifflichkeiten in der Literatur oftmals synonym verwendet werden [16]. Es finden sich Definitionen, die Engagement etwa als „engagement as usage“ oder als „effective engagement“ („Einbindung als Nutzungsverhalten“ vs. „effektive Einbindung“; [21]) bezeichnen. Bei dieser Definition wird, ähnlich wie bei der Adhärenz, die Perspektive der effektiven Anwendung im Sinne des „use as it is designed“ oder „intended use“ in den Mittelpunkt gestellt [22]. In einer frühen Definition wird Engagement als „… a quality of user experiences with technology that is characterized by challenge, aesthetic and sensory appeal, feedback, novelty, interactivity, perceived control and time, awareness, motivation, interest, and affect“, beschrieben [23]. In dieser Definition werden die Qualität der Erfahrung der Nutzer*innen sowie die ästhetischen und sensorischen Anreize bei der Anwendung in den Mittelpunkt gestellt. Aus Perspektive der Mensch-Computer-Interaktion wird Engagement als „... the subjective experience of flow, a mental state characterized by focused attention and enjoyment“ [22] definiert (sinng. „… die subjektive Erfahrung des Fluss‑/Stromerlebens, eines mentalen Zustands, der durch konzentrierte Aufmerksamkeit und Genuss gekennzeichnet ist“). Dies kann auch als „engagement as flow“ (sinng. „Einbindung als Fluss‑/Stromerleben“) umschrieben werden. Die Verhaltenswissenschaft hingegen charakterisiert Engagement als „... temporal patterns (e.g. frequency, duration and depth, e.g. use of specific intervention content) of usage“ (sinng. „… zeitliche Muster (z. B. Häufigkeit, Dauer) und Tiefe (z. B. Nutzung bestimmter Interventionsinhalte) der Anwendung“; [22]). Dies ist auch als „engagement as usage“ zu verstehen. Nach Barello et al. hat Engagement drei Dimensionen: (1) eine Dimension des Verhaltens (*Nutzungsverhalten*), (2) eine kognitive Dimension (*Denken und Wissen*) sowie (3) eine emotionale Dimension (*Gefühle und Emotionen*; [24]).

Es bleibt festzuhalten, dass sich die Ansätze „effective engagement“ und „engagement as usage“ auf der einen und „intended use“ oder „use as it is designed“ auf der anderen Seite nur schwer differenzieren lassen. Eine deutlichere Abgrenzung zur Adhärenz und zu den Definitionen des Engagements kann hingegen zu dem Ansatz des „engagement as flow“ vorgenommen werden. In den nachfolgenden Ausführungen werden für das Begriffsverständnis der Adhärenz bei digitalen Interventionen die drei Elemente nach Sieverink et al. zugrunde gelegt [2].

## Determinanten der Adhärenz bei digitalen Interventionen

Faktoren, die die Adhärenz beeinflussen, variieren je nach der gesundheitlichen Problemstellung, den Merkmalen der digitalen Intervention, den adressierten Outcomes, den Charakteristika der Nutzer*innen und dem Anwendungskontext [14, 19, 25]. In einer systematischen Übersichtsarbeit von Beatty et al. [26] identifizierten die Autor*innen Determinanten, die die Adhärenz bei digitalen Interventionen im Rahmen psychischer Krankheitsbilder, wie etwa Depressionen oder Angstzuständen, beeinflussen. Es wurden aus 1755 Publikationen nach Berücksichtigung der Ausschlusskriterien 36 Publikationen in die Analyse einbezogen. Die bedeutendsten Determinanten, die in den eingeschlossenen Studien quantitativ erfasst wurden, waren das weibliche Geschlecht, eine erhöhte Behandlungserwartung und das Angebot von persönlicher Unterstützung an die Nutzer*innen bei der Anwendung. Zeitmangel bei der Anwendung, die persönliche Unzufriedenheit mit den Interventionsinhalten sowie Interventionsinhalte, die von den Nutzer*innen als unpersönlich wahrgenommen werden, waren die stärksten Prädiktoren für Nichtadhärenz, die qualitativ erfasst wurden [26]. Ein weiterer Einflussfaktor ist der Zeitraum für die Anwendung der digitalen Intervention. Hierbei ist anzunehmen, dass mit zunehmender Dauer die Wahrscheinlichkeit für das Einstellen der Anwendung durch die Nutzer*innen ansteigt.

In einer systematischen Übersichtsarbeit von Arsenijevic et al. [19] untersuchten die Autor*innen, inwiefern die Merkmale der Gestaltung und der Implementierung digitaler Interventionen (Electronic Health Tools) zu einer besseren Adhärenz bei vulnerablen Bevölkerungsgruppen führen können. Solche Bevölkerungsgruppen umfassten hier ältere Menschen, Menschen mit niedrigem sozioökonomischen Status, Alleinerziehende sowie gesellschaftliche Minoritäten oder Menschen mit Migrationshintergrund. Aus 429 Publikationen identifizierten die Autor*innen 29 Studien, welche allerdings eine hohe Variabilität im Hinblick auf die Studiendesigns, Stichprobengröße, gesundheitliche Problemstellung, Merkmale der digitalen Interventionen, Charakteristika der Nutzer*innen sowie den Anwendungskontext aufwiesen. Ein zentrales Resümee der Autor*innen war, dass vulnerable Bevölkerungsgruppen eine geringere Adhärenz gegenüber den analysierten digitalen Interventionen aufweisen als nicht vulnerable Bevölkerungsgruppen. Die Ergebnisse weisen darauf hin, dass digitale Interventionen mit multimodalem Inhalt sowie einer direkten Interaktion und Unterstützung zwischen Nutzer*innen und den Anbieter*innen der Intervention zu einer höheren Adhärenz führen können. Jedoch ist die Diversität der Publikationen kritisch zu sehen.

Übergeordnet scheint einer der relevantesten Faktoren für die Adhärenz zu sein, inwieweit die Inhalte und das Design digitaler Interventionen auf die Bedürfnisse und Eigenschaften der Nutzer*innen abgestimmt sind [27]. Es ist davon auszugehen, dass, bei Nichtberücksichtigung der Bedürfnisse und Eigenschaften der Nutzer*innen, digitale Interventionen von diesen als nicht sinnvoll eingeschätzt werden und sie die Anwendung folglich reduzieren oder sogar in Gänze einstellen [28, 29].

## Evaluation und Messgrößen der Adhärenz bei digitalen Interventionen

Es besteht derzeit eine große Variabilität hinsichtlich der Anzahl und der Art der verwendeten Messgrößen zur Bestimmung der Adhärenz bei digitalen Interventionen. Da die Einflussfaktoren der Adhärenz sehr vielfältig sind [25], ist es notwendig, die Messgrößen je nach digitaler Intervention zu betrachten und anzupassen. Relevant ist hierbei, dass diese Messgrößen frühzeitig im Entwicklungsprozess ermittelt und im Falle einer Wirksamkeitsprüfung einer digitalen Intervention a priori definiert werden [25].

Oftmals werden als quantitative Messgrößen aggregierte Protokolldateien (Logs) oder Sensordaten [30] verwendet, um das Anwendungsverhalten der Nutzer*innen zu beschreiben [31]. Solche Protokolldateien lassen sich anhand der FITT-Kategorien definieren, was im Englischen für Frequency, Intensity, Time und Type (FITT) steht (Frequenz, Intensität, Zeit, Typ; [32]). Mit der *Frequenz* werden die Anzahl der Log-ins pro Nutzer*in beschrieben, die durchschnittlichen Log-ins in einer definierten Zeitspanne oder in der gesamten Dauer der Anwendung. Die *Intensität* beschreibt die absolute Anzahl oder den prozentualen Anteil der durch die Nutzer*innen aufgerufenen Interventionsinhalte oder der durchgeführten Aufgaben oder Module. Die *Zeit* wird beispielsweise dadurch gemessen, wie lange etwa der Besuch einer aufgerufenen Seite (z. B. Smartphone-App oder Webseite) pro Nutzer*in andauert oder durch die Anzahl der Tage, die zwischen dem ersten und letzten Log-in vergangen sind. Der *Typ* der Messgrößen beschreibt, wie oft unterschiedliche Inhalte, wie etwa ein Quiz, Gesundheitsinformationen oder das Eintragen von Vitalwerten, angewendet werden [32].

Die Auswahl der Messgrößen wird jedoch oftmals uneinheitlich vorgenommen. So identifizierten Sieverink et al. in ihrer Übersichtsarbeit eine Vielzahl unterschiedlicher Messgrößen, mit denen die Adhärenz in den 62 eingeschlossenen Studien gemessen wurde [2]. Dies waren die Anzahl von Anmeldungen/Sitzungen (*n* = 14), der Aufruf von Modulen (*n* = 30), die aufgerufenen und genutzten Funktionen (*n* = 16), die Anzahl der abgeschlossenen Übungen (*n* = 9), die Anzahl der angesehenen Seiten (*n* = 11), die Anzahl der Tage/Wochen/Monate (*n* = 19) oder der gesamte Zeitaufwand (*n* = 14) der Anwendung. In den Studien wurde jeweils eine unterschiedliche Anzahl von Messgrößen verwendet. In 32 der 62 Studien wurden lediglich eine, in 17 Studien 2, in 7 Studien 3 und in 6 Studien 4 oder mehr Messgrößen eingesetzt. Ähnliche Ergebnisse zu solchen Messgrößen finden sich in einer Übersichtsarbeit zum Thema Interventionen zur Gewichtsabnahme [33], einem Scoping-Review zum „effective engagement“ (s. oben) im Rahmen chronischer Krankheiten [34] sowie dem bereits benannten Review von Beatty et al. [26].

Kritisch ist hierbei jedoch zu berücksichtigen, dass, auch wenn sich durch Logs die Merkmale der Anwendung durch die Nutzer*innen quantitativ bestimmen lassen, die Daten nicht zwangsläufig etwas darüber aussagen, wie genau die Inhalte von den Nutzer*innen angewendet wurden [30]. Nutzer*innen könnten etwa edukative Inhalte einer Smartphone-App anklicken und öffnen, aber die Inhalte nicht oder nur unzureichend lesen oder verstehen [35].

Aufgrund solcher Problemstellungen sind zur Bestimmung der Adhärenz qualitative Daten als sinnvoll einzuschätzen, um detaillierte Gründe für eine hohe oder geringe Adhärenz zu identifizieren [32]. Hierzu lassen sich etwa Methoden der empirischen Sozialforschung oder der Mensch-Technik-Interaktion einsetzen. Es finden sich in der Literatur keine klaren Aussagen, welche Methoden und Messgrößen als Mittel der Wahl anzusehen sind. Benannt werden in diesem Zusammenhang qualitative Interviews, Think-Aloud-Protokolle, bei denen die Nutzer*innen bei der Anwendung einer Technologie in einer Testsituation „laut denken“, oder Fokusgruppeninterviews [32]. Die Berücksichtigung dieser Daten erfolgt derzeit jedoch leider noch selten. In den hier angeführten Übersichtsarbeiten wurden lediglich in der Übersichtsarbeit von Beatty et al. 8 Studien identifiziert, in denen qualitative Daten als Messgröße der Adhärenz herangezogen wurden [26]. Da Adhärenz als ein multidimensionales Konzept zu verstehen ist und von einer Vielzahl unterschiedlicher Faktoren abhängt, erscheint eine Reduzierung von Messgrößen der Adhärenz auf lediglich rein quantitative oder rein qualitative Daten als nicht ausreichend [2]. Infolgedessen erscheinen Mixed-Methods-Ansätze, in denen sowohl qualitative als auch quantitative Daten herangezogen werden, sinnvoll.

## Dosis-Wirkungs-Beziehung digitaler Interventionen

Studien weisen in verschiedenen Anwendungskontexten darauf hin, dass eine Dosis-Wirkungs-Beziehung zwischen der Intensität der Anwendung und den gemessenen Outcomes besteht [14]. Ähnliches berichten Donkin et al. [17] in einer Studie zu einem Onlinetherapieprogramm mit dem Ziel der Reduktion von Symptomen bei Menschen mit Depression mit einer Interventionsdauer von 12 Wochen. Bei den Personen, die eine Verbesserung der Symptome nach Ende des Programms bemerkten, betrug die Gesamtanzahl der online verbrachten Minuten in dem Programm 351,1 min. Im Vergleich dazu verbrachten Personen, die später keine Verbesserung der Symptome benannten, nur 292,6 min in dem Programm [17].

Eine hohe Intensität der Anwendung muss jedoch nicht zwangsläufig mit einem besseren Outcome in Verbindung stehen. Denn obwohl in verschiedenen Studien eine Dosis-Wirkungs-Beziehung beobachtet werden kann, lassen sich auch Studien identifizieren, in denen eine solche Beziehung nicht gemessen wurde [11, 36]. Es finden sich ebenso Studien, in denen Wirkungen nachgewiesen wurden, obwohl die Nutzer*innen nicht alle digitalen Interventionselemente nutzen [2]. Es scheint folglich relevant, die richtige Dosis zu ermitteln, wie aus dem „Goldilocks-Prinzip“ abzuleiten ist: „Not too much. Not too little. Just right“ („Nicht zu viel. Nicht zu wenig. Genau richtig“; [30]).

Dies geht jedoch mit der Problematik einher, dass für verschiedene Anwendungsfälle nicht ausreichend belegt ist, wie hoch die eigentliche Dosis der intendierten Anwendung sein muss, um eine effektive Wirkung zu erzielen [5]. Auch für die Ermittlung der „richtigen“ Dosis digitaler Interventionen findet sich in der Literatur eine große Variabilität [2]. So wurde etwa in der Übersichtsarbeit von Sieverink et al. in 34 der 62 eingeschlossenen Studien die Adhärenz als „the more use, the better“, also je mehr, umso besser, beschrieben, wohingegen nur in 28 Studien ein Schwellenwert für das Erreichen einer positiven Wirkung beschrieben wurde [2].

In einer systematischen Übersichtsarbeit von Smith et al. [33] analysieren die Autor*innen die Dosis-Wirkungs-Beziehung zwischen der Anwendung und den Outcomes digitaler Interventionen zur Reduktion des Körpergewichtes. Aufgrund der Ausschlusskriterien wurden aus 234 Studien lediglich 5 berücksichtigt. Es wurden zahlreiche Messgrößen beschrieben, die in Studien zur Evaluation der Dosis-Wirkungs-Beziehung herangezogen wurden. Hierzu zählen die Frequenz und Häufigkeit, mit der sich die Nutzer*innen in das Programm eingeloggten, die Dauer des Log-ins oder der prozentuale Anteil der angewendeten Interventionselemente. Die Autor*innen heben hervor, dass in nahezu allen Studien, die in die Übersichtsarbeit eingeschlossen wurden, eine Assoziation zwischen der Steigerung der Anwendung und den gemessenen Outcomes in der Studie bestand [33]. Die in den Studien adressierten Outcomes waren Wiederzunahme des Gewichts, Zufriedenheit mit dem Fitnesslevel, Gewichtsabnahme, Body-Mass-Index oder Hüftumfang. Es finden sich darüber hinaus weitere Studien, die eine Dosis-Wirkungs-Beziehung bei digitalen Interventionen zur Gewichtsabnahme indizieren [37].

## Verbesserung der Adhärenz bei digitalen Interventionen

Es stehen verschiedene Ansätze zur Verfügung, um die Adhärenz bei digitalen Interventionen zu verbessern. Auch hier ist es aufgrund der Vielzahl unterschiedlicher Einflussfaktoren nicht einfach, allgemeingültige Aussagen zu treffen [35]. Es ist hierbei jedoch notwendig, die Perspektive der Entwicklung digitaler Interventionen noch vor der Evaluation zu berücksichtigen.

Eine der wichtigsten Maßnahmen, die in diesem Zusammenhang getroffen werden muss, ist der frühzeitige Einbezug der Nutzer*innen in den Entwicklungsprozess digitaler Interventionen [19, 36]. Ein partizipativer Entwicklungsprozess stellt eine der wesentlichen Voraussetzungen dar, um Technologien zu entwickeln, die die Bedürfnisse und Eigenschaften der Nutzer*innen berücksichtigen, damit die Intervention als sinnvoll eingeschätzt wird. Im Rahmen der partizipativen Entwicklung können gängige Modelle der Technikakzeptanz herangezogen werden, beispielsweise die Unified Theory of Acceptance and Use of Technology (UTAUT) oder das Technology Acceptance Model (TAM). Durch solche Modelle lassen sich Faktoren identifizieren, die die Intention der Nutzer*innen, eine Technologie anzuwenden, direkt (z. B. durch Einfachheit der Bedienung, erwarteten Nutzen oder sozialen Einfluss) oder indirekt (z. B. durch Freiwilligkeit der Nutzung oder Erfahrungen im Umgang mit Technologien) beeinflussen können [29, 38].

Werden solche Faktoren, die die Akzeptanz aus Perspektive der Nutzer*innen bestimmen, nicht in die Entwicklung mit einbezogen, kann die Situation entstehen, dass die Adhärenz abnimmt. Studien konnten zeigen, dass die Gründe, warum Nutzer*innen die Anwendung einer digitalen Intervention im Bereich des Managements von Diabetes Typ II eingestellt haben, im Wesentlichen die frustrierenden Erfahrungen mit der Anwendung der Technologie waren sowie die Wahrnehmung, dass der Inhalt der Intervention irrelevant und unverständlich ist [39].

Überdies ist relevant, dass bei digitalen Interventionen zur Beeinflussung des Verhaltens der Nutzer*innen theoretische Konstrukte, wie etwa die sozialkognitive Theorie oder das transtheoretische Modell [40], verwendet werden. Eine Studie konnte zeigen, dass die Verwendung von Theorien zur Verhaltensänderung ein noch stärkerer Prädiktor der Adhärenz ist als etwa die Merkmale der Technologie [41]. In diesem Zusammenhang kann also nicht ausgelassen werden, vorbestehende Evidenz notwendiger Therapieinhalte in eine digitale Intervention einfließen zu lassen.

Des Weiteren weisen Ergebnisse verschiedener Studien und Übersichtsarbeiten darauf hin, dass die Integration von Spielelementen (Gamification) sowohl die Adhärenz als auch das Engagement („engagement as flow“) positiv beeinflussen können [42]. Unter Gamification wird verstanden, dass Spielelemente in nicht spielbezogenen Anwendungen verwendet werden. Spielelemente können etwa das Sammeln von Punkten und Abzeichen (Badges), Ranglisten, die Definition von Zielen und Leveln sowie weitere Belohnungselemente sein [42]. In einer randomisiert kontrollierten Studie wurde verglichen, inwieweit eine App mit Spielelementen und eine App ohne Spielelemente die Adhärenz bei der Anwendung positiv beeinflussen [43]. Die Ergebnisse indizieren, dass die Nutzer*innen, die die App mit Spielelementen angewendet haben, die App häufiger und intensiver einsetzten als die Nutzer*innen der App ohne Spielelemente [43].

Die bis hier beschriebenen Maßnahmen lassen sich vordergründig in frühen Phasen der Interventionsentwicklung verorten. Darüber hinaus lassen sich Maßnahmen zur Verbesserung der Adhärenz benennen, die bei der Durchführung von Evaluationsstudien angewendet werden können. So können sich persönliche (Face to Face) oder digitale Kontakte (z. B. per E‑Mail oder Forum) zwischen Nutzer*innen und den Studiendurchführenden positiv auf die Adhärenz auswirken, da so seitens der Nutzer*innen Probleme bei der Anwendung schneller kommuniziert werden können [44]. Hierauf weisen Studienergebnisse in verschiedenen Anwendungskontexten hin, wie etwa in Studien mit Anwendungen für vulnerable Bevölkerungsgruppen [19] oder für Patient*innen mit Diabetes Typ II [39]. Ein fehlender persönlicher Kontakt zwischen Nutzer*innen und Durchführenden einer Interventionsstudie kann dazu führen, dass Nutzer*innen nach der Reduktion der Adhärenz aus einer Studie komplett ausscheiden („dropout attrition“; [39]). Positiv wirkt sich auf die Adhärenz ebenfalls aus, wenn die Nutzer*innen eine monetäre Vergütung für eine Studienteilnahme erhalten [36].

## Ethische Betrachtung

Die hier vorgenommenen Erörterungen berühren Aspekte wie Datensicherheit, Datenschutz und ethische Fragestellungen. Da bei der Evaluation der Adhärenz das Verhalten der Nutzer*innen durch „logs“ sehr genau gemessen werden kann, berührt die Messung der Adhärenz deutlich die Privatsphäre der Nutzer*innen. Es zeigt sich, dass Nutzer*innen Unwohlsein verspüren, wenn andere Personen, in diesem Fall Forscher*innen, sehen können, wann sie welche Aktivitäten durchführen. Die (empfundene) Beeinträchtigung der Privatsphäre kann dazu führen, dass Nutzer*innen nicht alle Aktivitäten durchführen [26]. Folgerichtig gilt es bei der Bestimmung der Adhärenz durch quantitative Daten, das Schadenspotenzial und das Nutzenpotenzial in ein ausgewogenes Verhältnis zu bringen, um nicht notwendige Belastungen von den Nutzer*innen fernzuhalten [45]. Hierbei sind Forscher*innen angehalten sehr genau zu definieren, *welche* Parameter durch die Erfassung von Logfiles erhoben werden und für welchen Zweck. Folgerichtig sind für Nutzer*innen adäquate und verständliche Informationen bereitzustellen, damit sie befähigt werden, selbstbestimmte Entscheidungen zu treffen [46].

Es lässt sich derzeit ein Spannungsfeld zwischen Ethik und Nutzenbewertung ausmachen. Wie bereits beschrieben, ist für viele digitale Interventionen in frühen Stadien der Entwicklung nicht hinreichend klar, mit welchen Parametern die Adhärenz bestimmbar ist. Zudem ist nicht klar, welche Schwellenwerte gelten, um eine gute von einer schlechten Adhärenz zu unterscheiden. Dies unterstreicht auch aus ethischer Perspektive nochmals die Notwendigkeit, dass im Rahmen der Evaluation von digitalen Interventionen nicht lediglich auf das Prinzip „je mehr, umso besser“ gesetzt werden kann. Forscher*innen unterliegen in diesem Zusammenhang einer Begründungspflicht, fundierte Annahmen zu treffen, mit welchen Messgrößen die Adhärenz valide operationalisiert werden kann und welche Parameter hierzu notwendig sind. Überdies müssen diese fundierten Annahmen auch empirisch im Verlauf der Entwicklung und durch bestehende Evidenz begründet werden.

## Schlussbetrachtung

Ziel dieses Beitrags war es, Probleme in der aktuellen Forschungspraxis zum Konzept der Adhärenz bei digitalen Interventionen zu erörtern und die Bedeutung für eine bessere methodische Adressierung aufzuzeigen.

Grundsätzlich ist festzuhalten, dass theoretische und konzeptionelle Auseinandersetzungen mit der Adhärenz digitaler Interventionen von hoher Bedeutung sind. Derzeit wird diesem Sachverhalt in vielen Studien nicht genug Rechnung getragen. Dies gilt sowohl für die Verwendung der Begrifflichkeiten als auch für die Definition der Messgrößen und deren Erhebungsmethoden sowie der dazugehörigen Schwellenwerte zur Abgrenzung einer guten von einer schlechten Adhärenz. Der Ansatz des „je mehr, umso besser“ kann dabei nicht als hinreichend erachtet werden [2].

Es wird in Zukunft von Bedeutung sein, ein transparentes Reporting zur Entwicklung und Evaluation von Messgrößen der Adhärenz bei digitalen Interventionen zu etablieren. Dies ist sowohl von Relevanz, um die Beziehung zwischen digitalen Interventionen und Outcomes zu verstehen als auch um die Ergebnisse von Studien interpretieren und mit anderen Studien vergleichen zu können [35]. Insbesondere Prozessevaluationen, die begleitend zu Interventionsstudien durchgeführt werden, bieten das Potenzial, die Adhärenz spezifischer zu evaluieren. Die Evaluation der Adhärenz ist jedoch nicht nur im Rahmen der Konzeption und Durchführung von Interventionsstudien relevant, sondern sollte gleichermaßen nach der Implementierung berücksichtigt werden.
